# Analysis of Ageing Effect on Li-Polymer Batteries

**DOI:** 10.1155/2015/979321

**Published:** 2015-07-05

**Authors:** Simone Barcellona, Morris Brenna, Federica Foiadelli, Michela Longo, Luigi Piegari

**Affiliations:** ^1^Department of Electronics, Information and Bioengineering, Politecnico di Milano, 20133 Milano, Italy; ^2^Department of Energy, Politecnico di Milano, 20156 Milano, Italy

## Abstract

Lithium-ion batteries are a key technology for current and future energy storage in mobile and stationary application. In particular, they play an important role in the electrification of mobility and therefore the battery lifetime prediction is a fundamental aspect for successful market introduction. Numerous studies developed ageing models capable of predicting battery life span. Most of the previous works compared the effect of the ageing factors to a battery's cycle life. These cycles are identical, which is not the case for electric vehicles applications. Indeed, most of the available information is based on results from laboratory testing, under very controlled environments, and using ageing protocols, which may not correctly reflect the actual utilization. For this reason, it is important to link the effect of duty cycles with the ageing of the batteries. This paper proposes a simple method to investigate the effect of the duty cycle on the batteries lifetime through tests performed on different cells for different kinds of cycle. In this way, a generic complex cycle can be seen as a composition of elemental cycles by means of Rainflow procedures. Consequently, the ageing due to any cycle can be estimated starting from the knowledge of simpler cycles.

## 1. Introduction

Energy storage systems, usually batteries, are essential for electric drive vehicles, such as hybrid electric vehicles (HEVs), plug-in hybrid electric vehicles (PHEVs), and all-electric vehicles (EVs) [1]. However, the growing attention to energy aspects that leads to the introduction of renewable sources into a new concept of active electric grids makes storage systems one of the most important and critical research topics of the sector.

Many factors are affecting the operational characteristics, capacity, energy output, and performance of a battery. Different manufacturers have dissimilar approaches to address these issues by different chemical systems, additives, form factors, and dimensions, which will have significant effect to different performances and usages of the batteries [2]. In addition, many automakers have adopted Lithium-ion (Li-ion) batteries as the preferred electric drive vehicles (EDVs) energy storage option, capable of delivering the required energy and power density in a relatively small, lightweight package.

In fact, Li-ion batteries are a key technology for current and future energy storage, whether they are used for mobile or stationary application [3]. They are preferred over other battery technologies since they provide one of the best energy-to-weight ratios, have no memory effect, and have a slow self-discharge [4]. Recently, Li-ion batteries are applied to many sectors, as automotive, aerospace, or defense, due to their high energy density [5]. In particular, in the automotive sector the increasing demand for electric vehicles has forced consideration of other promising battery technologies, such as Li-ion batteries, to replace existing lead-acid batteries. However, this replacement is challenging due to the large power and energy demands placed on such batteries, while guaranteeing their safe operation. So the battery's capacity and performance are crucial for the abovementioned applications. Moreover, Li-ion battery cannot be overcharged and overdischarged because these conditions could damage it.

In this way, a battery management system (BMS) is essential for the system to get accurate knowledge of Li-ion battery's operation condition. In particular, a core function is to provide accurate estimates of state of charge (SOC) and state of health (SOH) of batteries. Many methods, among which Coulomb counting [6], open circuit voltage monitoring [7], impedance spectroscopy [8], fuzzy logic, [9], neural network [10], and Kalman filter [11–13], were proposed in order to estimate the battery SOC and SOH. All these methods with advantages and disadvantages are summarized in [14].

The extensive use of batteries in hybrid electric vehicles (HEVs) today requires establishing an accurate model of battery ageing and life. Battery ageing can be dissociated into two parts: the calendar ageing and the cycle one [15]. The calendar ageing corresponds to the irreversible proportion of lost capacity during storage. In other terms, it is the degradation caused by the battery storage [16, 17]. On the contrary, cycle ageing is associated with the impact of battery utilization periods called cycles (charge or discharge). It happens when the battery is either in charge or in discharge. This is a direct consequence of the level, the utilization mode, the temperature conditions, and the current solicitations of the battery. As a consequence, during a battery's lifetime, its performance slowly deteriorates because of the degradation of its electrochemical constituents which in turn results in the deterioration of the HEV performance and fuel efficiency. These undesirable effects include the loss of rated capacity, faster temperature rise during operation, less charge acceptance, higher internal resistance, lower voltage, and more frequent self-discharge. The most drastic effect is the loss of rated capacity [18].

For these reasons, identifying ageing and degradation mechanisms in a battery is the main and most challenging goal. Such processes are complicated as many factors from environment or from utilization mode interact to generate different ageing effects.

To get insight into these performances degradation, research efforts were dedicated to model Li-ion ageing, namely, capacity loss or impedance increase, and quantify the impact of ageing factors [19, 20]. In spite of intensive investigations on various positive and negative electrode chemistries, these ageing phenomena are not yet well understood and not quantified, and the combined impacts of temperature (T), SOC, depth of discharge (DOD), and current intensity still remain difficult to quantify and manage. Although most of Li-ion ageing mechanisms have been experimentally identified and described in the literature, these phenomena are complex and can interact with each other, resulting in different evolution shapes for capacity loss and power fade [21]. Such aspects are important over the lifetime of the vehicle and have been extensively studied through long-term experiments on various battery technologies. However, most of the available information is based on results from laboratory testing, under very controlled environments, and using ageing protocols, which may not correctly reflect the actual vehicle utilization. Also the ageing data provided by the battery manufacturers result from standard ageing tests, in which the battery is discharged and charged thousands of times with identical current profiles (or cycles). Therefore, significant differences in ageing and battery life may exist when the batteries are utilized on a vehicle under specific operating conditions and usage patterns, leading to conservative vehicle designs where the battery is typically oversized to guarantee performance and range near the end of life [22].

The problem of the effect of the duty cycle on batteries lifetime is not a trivial problem. In normal use, the batteries follow a power request that is never a standard discharge up to a fixed SOC. For this reason, it is very important to link the effect of duty cycles with the ageing of the batteries trying to make this complex problem as simple as possible.

In this paper, only the cycle ageing of Lithium-ion batteries is analyzed. A simple method to investigate on the effect of the duty cycle on the batteries lifetime, through tests performed on different cells, has been proposed. Through this method, it is possible to estimate the ageing of the batteries for different cycles starting from the knowledge of few parameters. A generic complex cycle can be seen as a composition of elemental cycles by means of Rainflow procedures [23]. In this way, the ageing due to any also complex cycle can be estimated starting from the knowledge of simpler cycles.

## 2. Experimental Test Procedure

The problem of evaluating the lifetime of batteries is a very hard task up to now not solved. In particular, for EVs, the information of the batteries SOH, together with that of SOC, could be very interesting.

As is well known, the life of batteries depends on many parameters: temperature, current, depth of discharge, voltage, humidity, and so on. Anyway, to take into account all these parameters is a very hard challenge. In this paper, the effect of the duty cycle on the batteries lifetime has been addressed.

The problem of the effect of the duty cycle on lifetime of batteries is a difficult problem. Indeed, the life of batteries is always given by manufacturers with reference to fixed cycles. They refer to discharge up to SOC values (usually 20% and 80% of the depth of discharge). In normal use, the batteries follow a power request that is never a standard discharge up to a fixed SOC. For this reason, it is very important to link the effect of duty cycles with the ageing of the batteries. In this paper, it would be shown that, in some conditions, it is possible to estimate the ageing of the batteries for different cycles starting from the knowledge of few parameters. In order to do it, the battery will be cycled with 2* elemental* cycles and then with a cycle composed of the first two. Then, the results will be analyzed to understand if the ageing of the composed cycle can be derived from the ageing of the elemental ones. If this is true, any cycle can be decomposed in elemental cycles by means of Rainflow procedures [23] and the ageing for any cycle can be estimated.

In order to evaluate the SOH of the batteries under tests, it has been chosen to consider their capacity as the indicator of SOH. For this reason, during the ageing tests, the batteries are periodically full charged and full discharged for measuring their capacity. Another indicator of SOH could be the internal resistance. For the approach used by the authors, information about internal resistance is considered only as an instrument for a SOH evaluator but is not used for* measuring* the state of ageing. With the aim of verifying the effect of duty cycles on the ageing process of batteries, the tests have been made with noncritical conditions for all the other variables. For this reason, the cells have been cycled in the following conditions:

- under ambient temperature and humidity (the temperature cell has been monitored);
- with a current lower than the rated current (C1) for avoiding ageing due to high currents;
- in a SOC region between 80% and 20% in order to avoid ageing occurring in the zones of high or low voltage.

The cell used for the test is a polymer Li-ion battery 8773160K manufactured by General Electronics Battery Co., Ltd. The main data of the cell are reported in Table 1.

**Figure 1:**
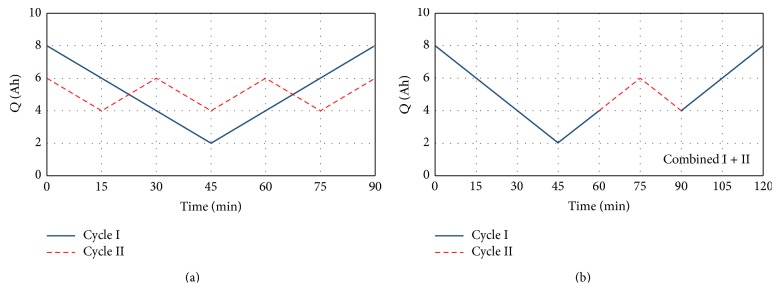
(a) Elemental test cycles and (b) combination of the two elemental cycles.

The two elemental cycles are defined as follows:

1. starting from a SOC equal to 80%, discharge 6 Ah (60%) at 8 A, and charge 6 Ah at 8 A,
2. starting from a SOC equal to 60%, discharge 2 Ah (20%) at 8 A, and charge 2 Ah at 8 A,
3. the third cycle, composed using the first two, which is constituted by charge and discharge phases at 8 A following the profile reported in Figure 1(b).

Figure 1(a) shows the two elemental tests defined in the above (1) and (2), while in Figure 1(b) the combination of the two elemental cycles is reported.

**Figure 2:**
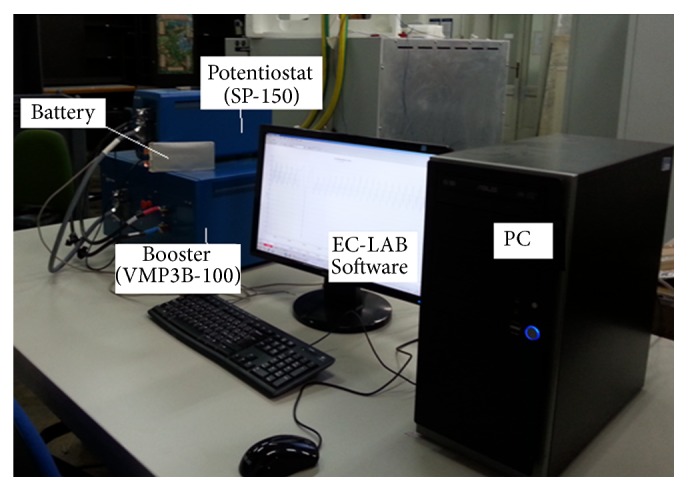
Experimental setup.

The three cycles have been applied to three fresh cells and, every 15 cycles, a full charge and discharge cycle has been performed for measuring the capacity of the cell. The tests have been performed at the Department of Electronics, Information and Bioengineering of the Politecnico di Milano using a 100-A booster (VMP3B-100) connected to a potentiostat (SP-150), which were both from Biologic Science Instruments, controlled by a PC via USB with EC-LAB software.  Figure 2 shows the experimental setup.

If the age effect of the third cycle can be obtained as a combination of the ageing effects of the first two cycles, it is possible to estimate the ageing of any cycle decomposing it in elemental cycles. The decomposition could be obtained, for example, by using Rainflow algorithms. The application of Rainflow techniques to batteries ageing is out of the scope of this paper in which only the possibility of estimating the ageing as a “sum” of ageing of elemental cycles will be addressed.

## 3. Experimental Results

As discussed in the previous section, the three cells have been tested with the cycles defined in Figure 1. At first, 300 cycles have been performed. Then, it has been verified that 300 cycles of kind 2 are too few for appreciating the ageing of batteries. Therefore, other 300 cycles, for a total of 600 cycles, have been performed on each cell. In the following, the three cells will be indicated with subscripts 1, 2, and 3 corresponding to the cycle to which they have been tested.

The three cells are, of course, different from one another. In order to compare their ageing, a preliminary measurement of their capacity has been performed and this value has been considered as reference value for evaluating the ageing. In particular, the initial measured capacity of the three cells is

(1) $$\begin{array}{c} C_{1}=10.191\;\text{Ah},\\ C_{2}=10.290\;\text{Ah},\\ C_{3}=10.268\;\text{Ah}. \end{array}$$

**Figure 3:**
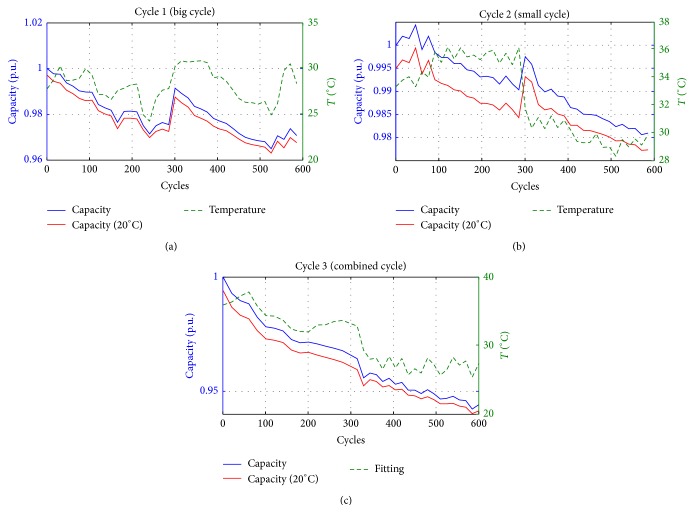
Ageing of the three cells as function of the cycles.

The following capacity reductions will be calculated per unit using the bases reported in (1). In Figure 3, the capacity reductions (blue line) of the three cells during the 600 cycles are reported.

Looking at the capacities obtained with the two elemental cycles a significant increasing at cycle 300 can be noted. This behavior is due to the pause introduced after 300 cycles. Indeed, as discussed above, initially 300 cycles have been programmed. Then, in order to increase the ageing effect, other 300 cycles have been performed. During the pause, the recovery effect of the batteries implies an increasing in the capacity for some cycles. In the combined cycle this effect is not visible because this cycle, being the last performed, has been executed without the pause. So, the first points after the pause should not be considered to analyze the ageing effect. In the behavior of the second cell, an increasing of the capacity after the first cycle can be observed. This could be due to effect of initial conditioning of the cell. For this reason, the initial capacity *C*_2_ is probably higher than the first value. In Figure 3 also the cells' temperature is shown.

**Figure 4:**
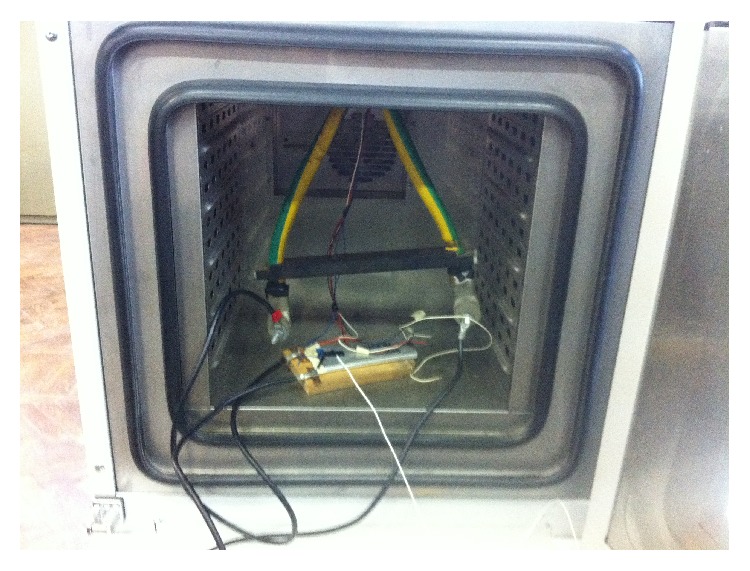
Climatic chamber.

Looking at Figure 3, it is possible to see that the temperature variation (green line) is limited in the range 25–40°C. This variation is due to the fact that the first set of 300 test cycles was performed during the summer while the other set was made during the autumn. As a consequence, it is important to report the different tests at the same temperature in order to obtain the battery ageing not depending on the temperature itself. In order to do that, it is necessary to know the variation of battery capacity as a function of the temperature. The latter relation is obtainable making other tests through which the capacity of the battery is measured at different temperatures using the climatic chamber reported in Figure 4.

**Figure 5:**
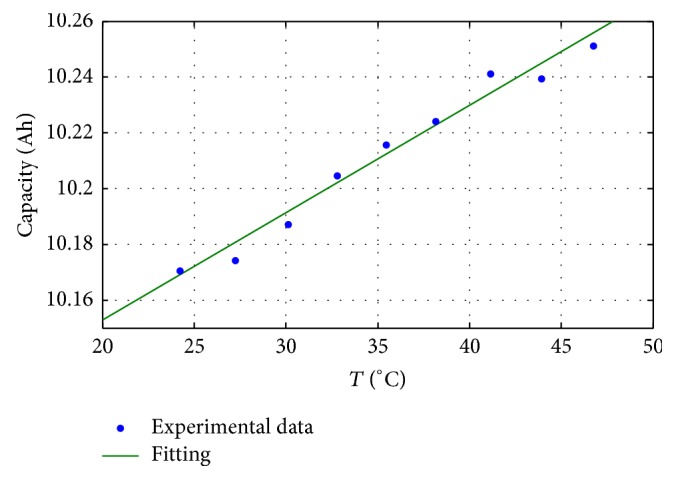
Battery capacity in function of the temperature.

So the battery capacity was measured in a temperature range of 20–40°C with a temperature step of 2.5°C, testing a new battery of the same kind. Test results are reported in Figure 5 together with a linear interpolated function.

The battery capacity as a function of the temperature, expressed in Ah (Figure 5), is as follows:

(2) $$\begin{array}{c} C\left(T\right)=0.003849T+10.08, \end{array}$$

where *T* is the temperature expressed in °C.

Figure 3 shows also the ageing of the three batteries reported at the same temperature of 20°C (red line) together with the ageing without the temperature correction (blue line). It is possible to note that the temperature effect is limited, about 1% of the battery capacity change with respect to a temperature variation of 20°C, as confirmed in [24–26].

**Figure 6:**
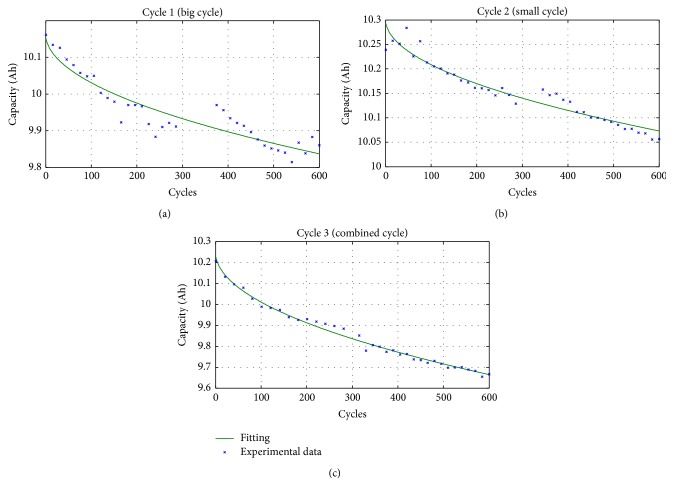
Interpolation of the ageing of the three cells.

Looking at the three behaviors of the battery ageing reported at the same temperature a linear decrease of the capacity with the square root of the number of cycles can be recognized. The interpolations, for the three curves, are reported in Figure 6 (green line) and the corresponding functions are the following:

(3) $$\begin{array}{c} C_{1}\left(n_{1}\right)=10.164-0.0134\sqrt{n_{1}},\\ C_{2}\left(n_{2}\right)=10.302-0.0093\sqrt{n_{2}},\\ C_{3}\left(n_{3}\right)=10.251-0.0239\sqrt{n_{3}}, \end{array}$$

where *n* is the cycle number and all the capacities are expressed in Ah.

It is worth to note that the ageing coefficient of the combined cycle is almost equal to the sum of the ageing coefficients of the two elemental cycles. Therefore, the ageing seems to be connected to the moved charge as stated for lead-acid batteries in [27] and, in particular, to the square root of the moved charge.

**Figure 7:**
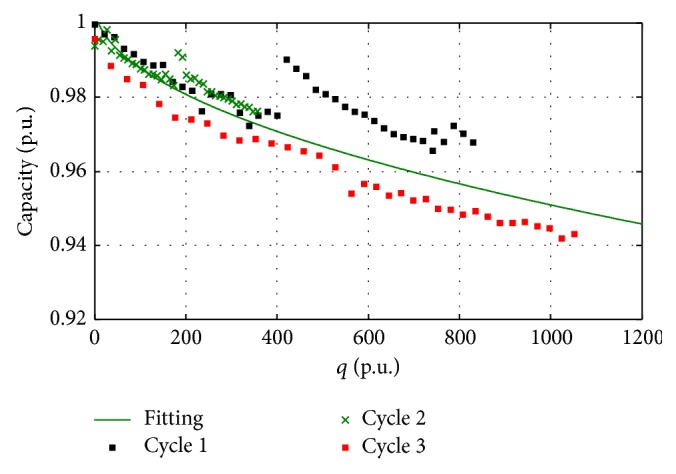
Ageing of the three batteries.

In order to understand if the effects of the two elemental cycles can be combined in the third cycle, (3) have to be expressed as function of the moved charge. Taking into account the moved charge of each cycle as reported in Figure 1, (3) can be rewritten as

(4) $$\begin{array}{c} C_{1}\left(q\right)=10.164-0.0035\sqrt{q}\;\left[\text{Ah}\right],\\ C_{2}\left(q\right)=10.302-0.0037\sqrt{q}\;\left[\text{Ah}\right],\\ C_{3}\left(q\right)=10.251-0.0056\sqrt{q}\;\left[\text{Ah}\right], \end{array}$$

where *q* is the charge, in Ah, moved by each battery. From (4) it is possible to see that the ageing is quite similar for the three batteries if the square root of the moved charge is considered as ageing factor. The coefficients of the two elemental cycles show a lower ageing in comparison with the combined test. This could be due to the pause after 300 cycles that has been inserted for the two elemental cycles and not for the combined cycle. With reference to the reported tests, moreover, it has to be taken into account that the* moved charge* is influenced also by the charge* moved* during capacity measurements (every 15 cycles). The capacities of the three cells as function of the square root of the moved charge are reported in Figure 7. In order to compare the residual capacities of the three cells, they was normalized on their initial rated capacity, while the moved charge was normalized on the nominal charge of the battery (10 Ah).

From the analysis of Figure 7, it can be stated that the ageing of the three batteries, measured as the reduction of their capacity, can be expressed, with good approximation, as function only of the square root of the moved charge. The interpolating function reported in the figure is

(5) $$\begin{array}{c} C\left(q\right)=1-0.0017\sqrt{q}\;\left[\text{p}.\text{u}.\right]. \end{array}$$

Looking at Figure 7 it can be seen that the recovery effect causes an increasing in the shown capacity with respect to the other data. In particular, the black circles, referring to the big cycle, after the pause occurring at approximately 4000 Ah (400 × 10 Ah), are quite higher than the interpolating function.

According to (5) the useful life of this kind of batteries could be predicted. Considering the end of life for the batteries when a reduction of the capacity of 20% is reached [28], from (5) a total charge of 160 kAh can be moved. Then, it is possible to state that the duty cycle does not practically affect the battery ageing.

It is worth to note that the life prediction does not take into account all the reactions that can occur when the voltage is close to the minimum or to the maximum but refers to the* best* use of the batteries in the* linear* region (i.e., where the voltage is a linear function of the SOC). Anyway, considering a cycle of 80% (i.e., 16 Ah, 8 for discharge and 8 for charge) the predicted life results are equal to 10000 cycles that is higher than the data given by manufacturers for this kind of battery. A change in degradation of the battery after a high number of cycles can be foreseeable. The prosecution of the test campaign will investigate this issue.

## 4. Conclusions

Many of the modern appliances, from mobile phones to electric cars, employ Li-ion batteries as their source of energy, so their correct operations depend on the actual performances of the battery. Therefore, it is important to estimate the health and the life of the battery considering different applications. Various models have been proposed in scientific literature to estimate the ageing of the batteries. Some of them consider chemical models that need many and often unknown parameters. Often the equivalent circuit models are preferred because of the easiest way to obtain the values of the parameters.

Battery models are essential for any battery-powered system design that aims at extending the battery's expected life and in battery power management.

For this reason, identifying ageing and degradation mechanisms in a battery is the main and most challenging goal. Such processes are complicated as many factors from environment or from utilization mode interact to generate different ageing effects.

Most of the available information concerning the battery ageing is based on results from laboratory testing, under very controlled environments, and using ageing protocols, which may not correctly reflect the actual vehicle utilization. Also the ageing data provided by the battery manufacturers result from standard ageing tests, in which the battery is discharged and charged thousands of times with identical current profiles (or cycles).

The problem of the effect of the duty cycle on batteries lifetime is important to estimate in a correct way the ageing of batteries. In normal use, the batteries follow a power request that is never a standard discharge up to a fixed SOC. For this reason, it is very important to link the effect of duty cycles with the ageing of the batteries.

This paper proposes a novel and simple way, but at the same time efficient, to estimate the life of the battery taking into account the effect of the duty cycle starting from the knowledge of few parameters that can be determined from reproducible tests. In particular, a generic complex cycle can be seen as a composition of different elemental cycles. In this way, the ageing due to any also complex cycle can be estimated starting from the knowledge of simpler cycles.

To this purpose many experimental tests have been carried out on three Li-ion batteries stressed with different charge/discharge cycles in order to estimate their state of health and consequently the effects due to their ageing.

The results, obtained from the analysis of the collected data, show that the ageing of the Li-ion battery is correlated with the total electric charge that flows into the battery cells and in particular with the square root of the total moved charge. Then, it is possible to state that the duty cycle does not practically affect the battery ageing. It is worth to note that the life prediction does not take into account all the reactions that can occur when the voltage is close to the minimum or to the maximum but refers to the best use of the batteries in the linear region. In addition, a change in degradation of the battery after a high number of cycles can be foreseeable. Therefore, further tests are needed to investigate this issue.

## Figures and Tables

**Table 1:** Main data of the cells under test.

Item	Specifications	Remark
Rated capacity	10000 mAh	0.2 C-5 A discharge, 25°C
Rated voltage	3.7 V	Average voltage at 0.2 C-5 A discharge
Standard charge current	0.2 C-5 A	Working temperature: 0~40°C
Max charge current	1 C-5 A	Working temperature: 0~40°C
Charge cut-off voltage	4.2 V	CC/CV
Discharge current	Continuously, 10 C; max, 15 C	Working temperature: 0~60°C
Discharge cut-off voltage	2.75 V	
Cell voltage	3.7–3.9 V	When leaving factory
Impedance	≤12 mΩ	AC 1 kHz after 50% charge, 25°C
Weight	Approx. 228 g	
Storage temperature		
≤1 month	−10~45°C	Best 20 ± 5°C for long-time storage
≤3 months	0~30°C	
≤6 months	20 ± 5°C	
Storage humidity	65 ± 20% RH	
